# From Core Facility to Collaborative Partner: Reimagining Institutional Shared Resources as CRO-Model Research Partners Enhanced by Agentic AI

**DOI:** 10.7171/001c.167753

**Published:** 2026-09-11

**Authors:** Jay W. Fox, Nicholas P. Ambulos

**Affiliations:** 1 Department of Microbiology, Immunology and Cancer Biology University of Virginia; 2 Genomics University of Maryland Marlene and Stewart Greenebaum Comprehensive Cancer Center

**Keywords:** Agentic AI, Bioinformatics, Contract Research Organizations, Core facilities, Shared Research Resources, Reproducibility, Biomedical Research

## Abstract

Academic biomedical research institutions are confronting a converging challenge: tightening federal research budgets and growing demands for faster and more reproducible science,1 and increasing pressure to maximize value derived from every research dollar. Traditional shared resources have enabled access to sophisticated technologies and expertise as transactional, fee-for-service facilities that prioritize throughput and cost recovery over scientific outcomes. We propose a shift toward a Contract Research Organization (CRO)-inspired operating model in which shared resources function as integrated research partners, supported by agentic artificial intelligence (AI) systems that enhance project management, experimental execution, data analysis, and scientific decision-making. This Perspective outlines the limitations of the current paradigm, identifies lessons from commercial CROs, explores the opportunities for agentic AI across major research domains, and presents a roadmap for institutional implementation. We argue that institutions that embrace this transformation will be better positioned to increase productivity, improve reproducibility, accelerate discovery, and strengthen research competitiveness in an increasingly resource-constrained environment.

## Introduction

Biomedical research in the United States stands at a critical inflection point. Federal investment through the National Institutes of Health (NIH) has remained largely flat or declined in real purchasing power over much of the past two decades when adjusted for biomedical inflation.^1^ Extramural grant success rates have dropped to well below 20% for many NIH institutes, personnel and scientific reagent costs continue to escalate, and equipment costs associated with emerging technologies are prohibitive for many individual laboratories.^2^ These financial pressures intersect with equally consequential scientific demands. Persistent concerns about reproducibility underscored by estimates that between 50% and 89% of published preclinical findings may not be replicable,^3,4^ which have intensified calls for more rigorous and transparent research practices. At the same time, expectations for faster translation of discoveries into clinical impact continue to grow, which creates pressure to accelerate research while maintaining scientific rigor.

In this environment, institutional shared resources including genomics, imaging, proteomics, and bioinformatics cores represent a substantial but underexploited strategic asset that is uniquely positioned to improve research efficiency, reproducibility, and scientific impact at scale. When operated effectively, shared resources provide substantial economies of scale, aggregate specialized expertise, and strengthen the research enterprise through workforce training, technology dissemination, and methodological standardization. Yet, their impact is constrained by persistent structural limitations: fee-for-service models that prioritize throughput over scientific outcomes, limited project ownership of research success by core personnel, fragmented operations that require investigators to navigate multiple vendor-like relationships, and inconsistent standards for quality, project management, and turnaround time. Many of these constraints are reinforced by institutional policies and cultural norms that favor transactional service delivery over scientific partnership. Paradoxically, these structures persist despite widespread recognition that deeper collaboration improves both efficiency and research outcomes.

Agentic artificial intelligence (AI) systems are AI systems capable of autonomously planning, reasoning, and executing multistep tasks with minimal human intervention, thus going beyond simple question-answering to take actions, use tools, call external services, and dynamically adapt to achieve a defined goal. Whereas traditional AI systems respond to a single prompt, agentic systems can break complex objectives into subtasks, iterate on results, and chain together workflows across diverse data sources and applications. Biomedical shared resources, including core facilities, biobanks, genomics centers, and clinical research infrastructure, represent a particularly promising environment for deploying agentic AI. These platforms generate complex workflows, integrate diverse data streams, and coordinate multiple stakeholders, thus providing opportunities for AI to enhance project management, operational efficiency, data quality, and scientific productivity. Such systems can autonomously manage sample intake and tracking workflows, intelligently query multiomics databases to generate relevant datasets for investigators, coordinate scheduling and resource allocation across technology platforms and personnel, generate and refine analysis pipelines in response to experimental parameters, assist with regulatory and compliance documentation, and proactively flag quality control issues in real time. By acting as an intelligent orchestration layer across projects, data streams, and stakeholders, agentic AI can reduce administrative burden, accelerate research timelines, and enable seamless integration across previously siloed biomedical workflows. This approach can elevate the role of shared resources from transactional service providers to integrated scientific partners, which increases their ability to deliver rigorous, reproducible, and high-impact science at an institutional scale.

This perspective outlines the evolution of institutional shared resources toward a contract research organization (CRO)-inspired model that is enhanced by agentic AI applications. We argue that this transformation, while requiring targeted investment in personnel, technology, and governance, offers a pathway to a competitive advantage. Institutions that successfully implement this strategic evolution will be better positioned to enable more rigorous science, accelerate discovery, strengthen grant applications, and maximize the impact of scarce research funding. Crucially, agentic AI is not an adjunct to this vision; it is the enabling layer that makes integrated CRO-like scientific partnerships operationally feasible across the scale and complexity of modern academic team science.

## Limitations of the Traditional Core Model

The established institutional model for operating shared resources was designed in an era of more abundant research funding, less intense grant competition, and, in some cases, lower demand for analytical throughput and reproducibility documentation. Six structural limitations define the current state and highlight the rationale for the proposed transformation.

First, a fee-for-service model can create misaligned incentives. Core facilities may optimize for billable instrument time rather than for scientific outcomes, while investigators may be charged for services regardless of whether the resulting data are ultimately usable. Given the potential ambiguity regarding ownership and responsibility for experimental design, sample acquisition, and sample preparation, there is a strong argument for establishing service agreements supported by a clearly defined Statement of Work. Second, limited project ownership often confines core staff to executing discrete technical tasks, reducing opportunities to engage with the broader scientific objectives, or contributing to experimental design and strategy. Third, the siloed structure of core facilities places the responsibility for crossfunctional coordination on principal investigators (PIs), creating inefficiencies and administrative overhead that intensify as projects incorporate increasingly complex experimental approaches. Fourth, most cores operate reactively, responding to emerging technologies, optimized experimental approaches, or cost-saving opportunities. Fifth, inconsistent quality metrics can create uncertainty in project planning and execution, which pose particular challenges for studies operating under strict grant-funded timelines. Sixth, poor coordination of analytical capability can generate downstream bottlenecks in individual laboratories, even when upstream sample processing is efficient.

## The Commercial CRO Model: Applicable Lessons for Academic Institutions

CROs emerged in the pharmaceutical and biotechnology industries to allow drug developers to outsource specific research and development functions while maintaining scientific quality and providing critical regulatory compliance. The CRO model is built on scientific partnership over transaction, project-based engagement with defined deliverables, crossfunctional coordination, and risk-sharing, with transparent and open communication with sponsors. These principles can be translated directly to the academic shared resource environment.

Academic cores can be aligned with four key elements of the CRO model. (1) Project-based scientific engagement replaces discrete service requests with engagements defined by scientific objectives and a coordinated plan for study execution. (2) Dedicated scientific project management (SPM) assigns a named PhD-level contact to each major engagement, providing investigators with a knowledgeable point of contact who understands the scientific objectives, communicates proactively, and adapts strategies when results diverge from expectations. (3) Integrated, multimodal service delivery enables seamless management of complex projects spanning genomics, bioinformatics, imaging, flow cytometry, and other disciplines through a single point of coordination, eliminating the need for PIs to manage multiple independent core relationships. (4) Analytical and interpretive support extends core engagement beyond data generation to include primary data analysis, helping to alleviate bottlenecks that often constrain progress within individual laboratories. Although there are shared resources throughout the country that have onboarded some of these elements, very few have embraced them at the institutional level.

Among all proposed changes, investment in PhD-level SPMs represents the single most powerful lever for driving organizational transformation. SPMs can serve as the intellectual bridge between the investigator’s scientific aims and the technical capabilities of the core. SPMs become the trusted scientific interface between investigators, core facilities, and emerging AI capabilities by serving as both relationship managers and quality gatekeepers and providing the human judgment, domain expertise, and accountability necessary to validate, interpret, and operationalize AI-generated recommendations in partnership with the PI.

## Agentic AI as the Enabling Layer

Agentic AI refers to AI systems capable of autonomous, multistep goal pursuit systems that can plan sequences of actions, invoke external tools or instruments, evaluate intermediate results, adjust experimental approaches, and iterate toward a defined objective without requiring step-by-step human instruction.^5^ The key distinction from conventional AI tools is significant: traditional AI answers a question, whereas agentic AI completes a workflow. This distinction is what makes agentic AI an appropriate enabling technology for CRO-model shared resources.

Across major research modalities, agentic AI offers transformative capabilities. In genomics and transcriptomics cores, agentic systems can monitor sequencing runs in real time, trigger quality control (QC) analysis upon completion, automatically flag low-quality libraries, and draft methods sections and data availability statements in compliance with current NIH and journal reporting requirements all without manual intervention. In advanced imaging cores, agentic systems interface with microscope control software to dynamically adjust acquisition parameters and apply deep learning–based segmentation to large image datasets to deliver structured morphometric and colocalization outputs. In flow cytometry, agentic AI applies spectral modeling to recommend optimized panel configurations and automates gating and population identification across multiparameter datasets. In proteomics, agentic pipelines manage data-dependent acquisition optimization, post-translational modification mapping, and multiomics integration in real time.

AI is particularly transformative in current scientific literature surveillance and study planning. Autonomous literature surveillance continuously monitors PubMed, bioRxiv, and relevant journals for new publications pertaining to active projects and delivers weekly digests with relevance rankings. Agentic platforms can support the development of Specific Aims narratives, Approach sections, and bioinformatics analysis plans from investigator-provided scientific objectives, insights, and preliminary data. Power analyses and experimental design recommendations can be generated from pilot data and literature comparisons, providing investigators with defensible sample size calculations at the early stages of project planning. These tools will find utility in both the management of shared resources and in individual investigators’ laboratories.

## Integration: The AI-Augmented Research Services Hub

The CRO-model transformation should not be organized at the level of individual cores but instead at the level of a research services hub asan institutional governance structure with a director, a faculty advisory committee, and a centralized AI governance layer. Institutions that have a centralized organization and management of shared resources are best poised for this integration. Allowing individual cores to adopt AI tools independently risks creating fragmentation, inconsistent practices, data governance gaps, and unclear accountability, which ultimately undermine the reproducibility, and standardization benefits that AI is intended to enhance. A centralized AI governance layer defines platforms, data pipelines, security standards, and audit requirements that apply across all shared resource operations, thus ensuring that AI-generated outputs are traceable, reproducible, and institutionally accountable.

Within this structure, the AI-augmented SPM is the operational cornerstone, integrating scientific expertise, project coordination, and AI-enabled decision support. Agentic AI addresses the fundamental scalability challenges of the SPM role. Without AI support, an SPM managing multiple complex, multimodal projects inevitably encounters capacity constraints. With AI augmentation, however, each SPM can manage substantially more concurrent projects potentially increasing their capacity by 30%–50% while maintaining analytical rigor and high-quality scientific engagement. The SPM, in partnership with the PI, defines scientific objectives, exercises judgment at critical decision points, and maintains investigator relationships. The agentic AI platform handles orchestration, monitoring, preliminary analysis, and the organization and preparation of deliverables.

## Tiered Engagement Model

**Figure 1.**
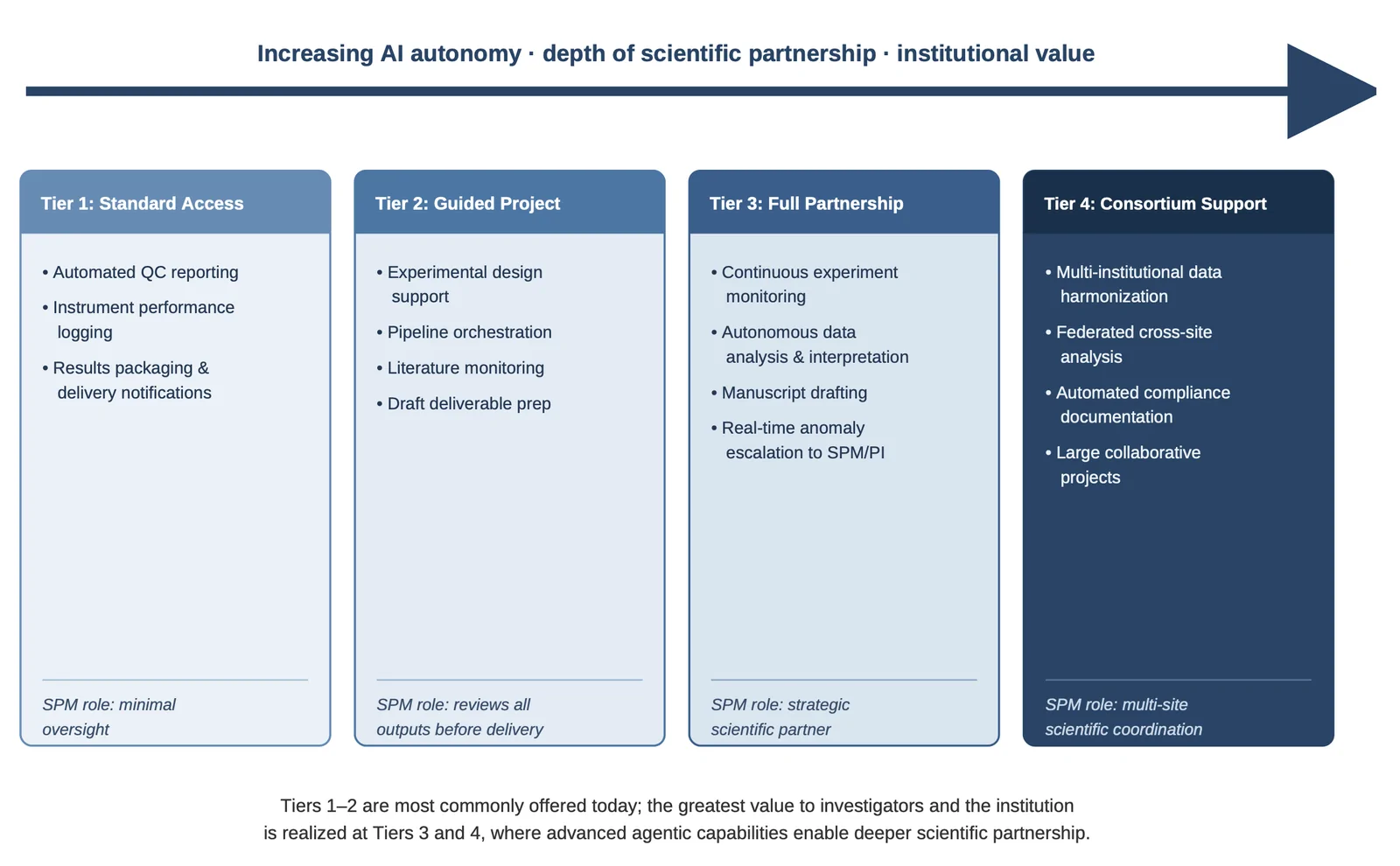
Tiered engagement model for an AI-augmented research services hub. Service is organized into four escalating tiers, ranging from largely automated support (Tier 1, Standard Access) to multi-institutional consortium support (Tier 4, Consortium Support). Moving across the tiers, the autonomy of agentic AI, the depth of scientific partnership, and the strategic value delivered to investigators and the institution all increase, while the role of the SPM shifts from minimal oversight to strategic scientific partnership. Tiers 1 and 2 are the most commonly offered today, whereas the greatest value to both the investigator and the institution is realized at Tiers 3 and 4.

Engagement can be structured in four tiers (Figure 1). At Tier 1 (Standard Access), agentic AI automates tasks including QC reporting, instrument performance logging, results packaging, and delivery notifications with minimal SPM oversight. At Tier 2 (Guided Project), AI assists in experimental design, pipeline orchestration, literature monitoring, and draft deliverable preparation with SPM review of all outputs before delivery to the investigator. At Tier 3 (Full Partnership), AI continuously monitors all active experiments, autonomously performs data analysis and interpretation, drafts manuscript sections, and escalates anomalies to the SPM and PI in real time. At Tier 4 (Consortium Support), multi-institutional data harmonization, federated analysis across partner sites, and automated compliance documentation are available for large collaborative projects. This tiered structure enables institutions to align service intensity with investigator needs and project complexity while also managing hub capacity efficiently. There are institutions offering one or more of Tiers 1–3, with Tiers 1 and 2 being most commonly provided. The greatest value to both the PI and the institution is realized at Tiers 3 and 4, where advanced agentic capabilities enable deeper scientific partnership, increase productivity, and accelerate discovery.

## Responsible AI Principles for Research Services

The integration of agentic AI into scientific workflows requires institutions to establish and enforce responsible AI principles. We propose six core principles for AI governance within research services hubs.

1. **Transparency:** All AI-generated analyses are disclosed to investigators. The specific tools, versions, and parameters used are documented in every deliverable.
2. **Human oversight:** No AI output is delivered to investigators without SPM review. The AI identifies and summarizes findings while the SPM applies scientific expertise to assess validity, significance, and appropriate interpretation.
3. **Reproducibility:** All AI-assisted analyses are conducted in version-controlled environments with complete parameter logs. Results must be fully reproducible from the documented inputs, methods, and configurations.
4. **Data governance:** AI systems operate within institutional data governance frameworks. Patient-derived and controlled-access datasets are handled only within approved security boundaries.
5. **Continuous validation:** AI tool performance is benchmarked regularly against expert human analysis. Performance degradation or distributional shift trigger human review and tool revalidation, with a target of at least 95% concordance with expert review.
6. **Equity:** AI-assisted services are available across all service tiers and to all investigators based on project needs. Service differentiation should reflect the level of engagement and support required, but it should not create disparities in the quality of analytical capabilities available to investigators.

These principles align with and extend the AI governance guidance increasingly promulgated by journals, funding agencies, and professional societies.^6^ Institutions adopting this model should anticipate that AI use in deliverables will require disclosure in publications analogous to the author disclosure requirements that are now standard at journals including The FASEB Journal and should establish templates and workflows to support investigators in meeting those requirements.

## Financial Considerations

The deployment of agentic AI requires substantial investments, including platform licensing or development, computational infrastructure, staff training, and ongoing governance overhead. However, the return on this investment can be equally substantial. Conservative modeling suggests that automated pipeline execution and report generation can replace an estimated 0.5–1.0 full-time equivalent of bioinformatics analyst effort per core annually ($75,000–$150,000 per core). Real-time QC gating that prevents low-quality samples from proceeding through costly downstream pipelines may prevent $50,000–$200,000 in failed experiments hub-wide each year, depending on the size of the enterprise. Also, AI-assisted methods development, power analyses, and bioinformatics planning can strengthen grant applications and improve funding competitiveness, potentially generating incremental awards of $500,000–$2 million or more for a mid-sized research institution. Thus, delivering potential returns that can substantially exceed the initial investment in AI platforms and personnel.^6^

## A Roadmap for Institutional Transition

We propose a four-phase roadmap for institutional implementation. Phase 1 (Months 1–6) is Assessment and Readiness: this phase establishes the foundation for implementation through a comprehensive assessment of shared resources, evaluation of AI readiness and governance capabilities, financial modeling of the hub framework, and stakeholder alignment across research and academic leadership. Phase 2 (Months 6–18) is Pilot Program Design and Launch: activities here include selecting two to four scientifically complementary pilot cores, hiring or redeploying two to three SPMs assigned to pilot investigator cohorts and prioritize early-career faculty and high-complexity multimodal projects, deploying one to two agentic AI tools with baseline performance benchmarking, and establishing key performance metrics, including investigator satisfaction, time to data delivery, cost per deliverable, and AI accuracy relative to expert validation. Phase 3 (Months 18–36) is Hub Formation and AI Platform Integration: this includes establishment of the research services hub governance structure, deploying centralized AI governance, and launching a faculty communication and change management program including transparent reporting on AI use in deliverables. Phase 4 (Years 3–5) is Strategic Capability Expansion: this final phase includes activation of AI-powered technology scouting, development of industry partnership capabilities, and pursuit of federated AI models enabling crossinstitutional data analysis without centralizing sensitive research data.

## Change Management and Anticipated Challenges

The single most common failure mode for shared resource transformation initiatives is faculty perception that centralization is being implemented to advance administrative efficiency rather than scientific excellence, which results in concerns about diminished investigator autonomy, reduced flexibility, and loss of control over the research process. This must be addressed at every stage with genuine faculty participation, transparent governance, and early evidence of improved scientific outcomes from pilot cohorts. Potential resistance from established PIs is best managed through phased, voluntary adoption, with engagement at Tier 2 and above remaining optional during the pilot phase. Additional momentum for change can be created by partnering with the shared resources to provide graduate students with hands-on training opportunities while they advance their own research projects.

One could envision the SPM role being fulfilled initially by the shared resource enterprise director, or perhaps by individual shared resource directors who maintain strong networks across institutional core facilities. However, achieving the full potential of the model will likely require the recruitment of additional PhD-level SPMs, supported by compensation packages that are competitive within the industry and accompanied by well-defined career advancement pathways. This workforce can be developed in partnership with graduate programs and strengthened through explicit opportunities for publication authorship, grant coauthorship, and professional development. Integration of disparate systems across cores will require prioritized investment in a unified platform during Phase 3, with strong interoperability requirements in platform selection. The risk of AI performance degradation over time should be mitigated through continuous benchmarking against expert human analysis, with predefined performance thresholds triggering review, recalibration, or retraining. Faculty skepticism about AI-assisted outputs can be effectively addressed through transparent documentation of data provenance, analytical workflows, and model performance, complemented by the early dissemination of validation studies demonstrating concordance between AI-generated and expert-reviewed results.

## Conclusion

The biomedical research enterprise is entering a period that will reward institutions and investigators who adapt their operating models to a more constrained, faster-moving, and higher-rigor environment. The emergence of mature, deployable agentic AI platforms makes the transformation of institutional shared resources from passive service providers to active, CRO-like scientific partners a strategic imperative and not a distant aspiration.

The question is not whether the academic research model needs to evolve; it does. Building CRO-inspired shared resource infrastructure enhanced by the responsible integration of agentic AI is a far more strategic approach than resorting to reactive cost-cutting in the middle of a funding crisis. Shared resources, reimagined and AI-augmented, are not a retreat from scientific ambition. They are a force multiplier for it. We invite the research community to engage with this framework, stress-test its assumptions, and contribute to the governance and validation standards that responsible AI adoption in academic science will require.

### Financial Support/Conflict of Interest {#fund}

The authors declare no conflicts of interest and there is no external funding.
